# Adult Hematology and Clinical Chemistry Laboratory Reference Ranges in a Zimbabwean Population

**DOI:** 10.1371/journal.pone.0165821

**Published:** 2016-11-03

**Authors:** Wadzanai P. Samaneka, Gibson Mandozana, Willard Tinago, Nehemiah Nhando, Nyaradzo M. Mgodi, Mutsawashe F. Bwakura-Dangarembizi, Marshall W. Munjoma, Zvenyika A. R. Gomo, Zvavahera M. Chirenje, James G. Hakim

**Affiliations:** 1 University of Zimbabwe-University of California San Francisco Collaborative Research Programme, Harare, Zimbabwe; 2 Department of Community Medicine, University of Zimbabwe College of Health Sciences, Harare, Zimbabwe; 3 Department of Pediatrics, University of Zimbabwe College of Health Sciences, Harare, Zimbabwe; 4 Department of Chemical Pathology, University of Zimbabwe College of Health Sciences, Harare, Zimbabwe; 5 Department of Medicine, University of Zimbabwe College of Health Sciences, Harare, Zimbabwe; 6 Department of Obstetrics and Gynecology and Reproductive Health Sciences, University of Zimbabwe College of Health Sciences, Harare, Zimbabwe; 7 University of Zimbabwe Clinical Research Centre, Harare, Zimbabwe; Oregon State University, UNITED STATES

## Abstract

**Background:**

Laboratory reference ranges used for clinical care and clinical trials in various laboratories in Zimbabwe were derived from textbooks and research studies conducted more than ten years ago. Periodic verification of these ranges is essential to track changes over time. The purpose of this study was to establish hematology and chemistry laboratory reference ranges using more rigorous methods.

**Methods:**

A community-based cross-sectional study was carried out in Harare, Chitungwiza, and Mutoko. A multistage sampling technique was used. Samples were transported from the field for analysis at the ISO15189 certified University of Zimbabwe-University of California San Francisco Central Research Laboratory. Hematology and clinical chemistry reference ranges lower and upper reference limits were estimated at the 2.5^th^ and 97.5^th^ percentiles respectively.

**Results:**

A total of 769 adults (54% males) aged 18 to 55 years were included in the analysis. Median age was 28 [IQR: 23–35] years. Males had significantly higher red cell counts, hemoglobin, hematocrit, and mean corpuscular hemoglobin compared to females. Females had higher white cell counts, platelets, absolute neutrophil counts, and absolute lymphocyte counts compared to males. There were no gender differences in eosinophils, monocytes, and absolute basophil count. Males had significantly higher levels of urea, sodium, potassium, calcium, creatinine, amylase, total protein, albumin and liver enzymes levels compared to females. Females had higher cholesterol and lipase compared with males. There are notable differences in the white cell counts, neutrophils, cholesterol, and creatinine kinase when compared with the currently used reference ranges.

**Conclusion:**

Data from this study provides new country specific reference ranges which should be immediately adopted for routine clinical care and accurate monitoring of adverse events in research studies.

## Background

Laboratory reference ranges are used to guide the interpretation of laboratory results and for the proper management of patients in clinical and research settings. Reference values are established from a sample of the reference population and selected on the basis of criteria such as age, gender, dietary habits, ethnicity, and the environment [1, 2, 3]. For the correct interpretation of laboratory results and safety of study participants, it is imperative that reference ranges are appropriate for the relevant population. A Nigerian study reported remarkable differences in normal reference ranges compared to those of other countries [4].

Laboratory reference ranges currently used in clinical and research settings in Zimbabwe such as the University of Zimbabwe-University of California San Francisco (UZ-UCSF) collaborative research program are derived from several sources, including textbooks and local studies of varying rigor, many of which are over a decade old [5, 6, 7, 8, 9, 10]. Textbook reference ranges are often based on Western populations and significant differences have been observed from those of African origin [1, 3]. The Clinical Laboratory and Standards Institute (CLSI) guidelines recommend that reference ranges be descriptive of a specific population and ideally are to be verified every 5 years [11].

This study was therefore conducted to determine current adult hematology and clinical chemistry reference ranges for a Zimbabwean population.

## Materials and Methods

### Study Settings

The UZ-UCSF is a Zimbabwe based United States National Institutes of Health (NIH) funded Clinical Trials Unit (CTU) which has been conducting Phase I/ II and III multicenter HIV clinical trials since 1994. Participants were derived from the general population within the UZ-UCSF research catchment areas of Harare, Chitungwiza, and Mutoko. Harare is the capital and largest city of Zimbabwe with a population of 1.5 million; Chitungwiza is a dormitory city for Harare with 350,000 inhabitants located 30 km southeast of the capital and Mutoko is a rural community of 40,000 inhabitants located 140 km northeast of Harare [12].

### Study Design

A cross-sectional study of a representative sample of the adult population of Harare, Chitungwiza, and rural Mutoko was used to determine reference ranges for hematology and chemistry parameters.

### Sampling Strategy

The administrative units in Zimbabwe comprise provinces, districts, and wards. In cities and towns, a ward is a division created for administrative or legislative purposes, while in rural areas, wards are comprised of villages. Community sensitization in the wards was conducted by experienced outreach workers prior to commencement of the study. Multistage sampling technique was used to select the areas from which participants were drawn. The first-stage sampling unit was the ward. We randomly selected 38 of 52 wards in Harare, 8 of 24 in Chitungwiza, and 3 of 8 in Mutoko. The second stage was household sampling, where households were systematically selected from a randomly selected reference point. After identifying a household, the head of the household or any other adult household representative described the household composition. A research assistant stratified household members according to age, which was verified by proof of national identification document. In households with more than one person of the same age group and/or sex, a Kish grid was used to randomly select the participant. However, in order to have approximately equal numbers of males and females, the research assistant alternately chose a male or female from one household to the next.

### Participants

This analysis is part of a larger study to determine Zimbabwean hematology and clinical chemistry laboratory ranges in children, adolescents and adults that was conducted between May and September 2011. A total of 2359 participants aged 0–55 years were recruited into the study; divided into pediatric (0-<12years), adolescent (12 -<18years) [13] and adult (18–55years) cohorts. This paper is based on the 769 adult cohort. Participants were healthy volunteers with no history, clinical or laboratory evidence of chronic disease (hypertension, diabetes, liver or renal disease) and were human immunodeficiency virus infection (HIV) and hepatitis B surface antigen (HBsAg) negative on laboratory testing. Participants were on no medications for acute illness (or were one week past completion of a course of medication) with no abnormal findings on clinical and laboratory examination.

### Ethical considerations

The protocol was approved by the Joint Research Ethics Committee of the University of Zimbabwe and Parirenyatwa Teaching Hospital and the Medical Research Council of Zimbabwe. Each participant provided a written informed consent.

### Specimen collection and handling

All samples were collected by a clinician using standard phlebotomy procedures as described elsewhere [13]. Blood was collected as follows: 10 mL in a plain tube for chemistries, 10 mL in a purple EDTA tube for full blood counts, and 4 mL in a grey sodium fluoride tube for glucose testing. The sodium fluoride tubes were immediately placed on ice pack at collection. All samples were transported to the UZ-UCSF Central Laboratory in temperature controlled bags maintained at 15–25°C and were processed on average 6 hours after sampling [13].

### Laboratory procedures

All samples were delivered to the ISO 15189 certified UZ-UCSF Central Laboratory on the day of collection. Processing was completed after specimen verification at the Central Laboratory’s specimen management section and all samples were analyzed on the day of collection. All samples were entered into the electronic database (DISA Laboratory Information system, Laboratory System Technologies, version 16.03).

### Hematology

With whole blood collected in the EDTA tube, white cell count, hemoglobin, automated five-part differential, platelet count, mean corpuscular volume (MCV), mean corpuscular hemoglobin concentration (MCHC), mean corpuscular hemoglobin (MCH), red blood cell count and hematocrit were determined using Sysmex XT2000I *(Symex Europe D-22848 Nordersted*, *Germany)* operated in the manual mode.

### Clinical chemistry and Serology

Using the blood sample collected in the plain tubes, biochemical analysis, HIV and Hepatitis B (HBV) screening were determined. Samples were centrifuged at 1100xg for 10 minutes and serum kept at 2–8°C pending testing. HIV screening was performed using Determine (Abbott Diagnostics, Wiesbaden, Germany) and oral quick (Orasure Technologies, Inc, Bethlehem, PA, USA). HBsAg was tested in serum using an enzyme immune assay manufactured by Bio-Rad Laboratory, (Redmond, WA, USA). Those testing HIV positive or HBsAg positive were excluded from the analysis. The chemistries assayed were: sodium, potassium, chloride, phosphate, creatinine, creatinine kinase, calcium, urea, aspartate amino transferase (AST), alanine amino transferase (ALT), alkaline phosphatase (ALP), albumin, total protein, gamma glutamyl transferase (GGT), total bilirubin, direct bilirubin, bicarbonate, triglycerides, lipase, cholesterol, high density lipoprotein-cholesterol (HDL-C), low density lipoprotein-cholesterol (LDL–C) and glucose. All tests were analyzed using the Hitachi 902 (*Hitachi*, *Roche diagnostics D-68292 Mannheim Germany*) according to manufacturer specifications. Methods for all analysis were traceable to International Federation of Clinical Chemistry (IFCC) standards.

### Quality Control

To perform quality control on the hematology measures, three levels of E-Check controls (low, normal and high level) (Sysmex Corporation 1-5-1 Wakinohama-Kaigandori Chuo-ku Kobe Japan) were run daily. Quality control for the biochemical assays was performed by running daily assays for two analytes, precinorm and precipath, (Roche Diagnostics GmbH, Sandhofer Strasse 116, and D-68305 Mannheim) prior to sample testing. Each analyte performance was tracked using real-time Levey Jennings QC plots monitored within 2 standard deviation of the laboratory established control mean. The UZ-UCSF Central laboratory also participates in External Quality Assurance (EQA) programs provided by the College of American Pathologists and monitored by Patient Safety Monitoring in International Laboratories (SMILE) for both Hematology and Clinical Chemistry. The EQA performance was acceptable for all panels processed over the study period

### Data Management and Statistical analyses

Demographic data were entered into a Microsoft Access 2007 database and laboratory data into DISA Laboratory Information System (Laboratory System Technologies, version 16.03). Demographic and laboratory data were merged and analyzed in STATA 12.1 (College Station, Texas, USA) and the Reference Value Advisor version 2.1 (Microsoft,Radmond,WA,USA). All calculations for determining reference intervals were based on the CLSI guidelines [11]. The Reference Value Advisor uses non-parametric statistical methods to determine the 95% reference limits, with the lower limit defined as the 2.5^th^ percentile and the upper limit as the 97.5^th^ percentile. Ninety percent confidence limits around the lower and upper confidence estimates were determined using a nonparametric bootstrap method, (not reported). Our underlying assumption was that the 2.5% and 97.5% centiles contain 95% of the distribution of normal values of the reference population. Extreme values were handled as described in the CLSI guidelines by performing a range check and the methods of Dixon (Dixon’s Test). Mann-Whitney U tests were used to analyze differences by gender. The reference intervals were calculated separately for men and women and then combined, both are reported. A two-sided p-value of <0.05 was considered significant.

### Participant safety

All results were reviewed by study clinicians. The study team communicated all results to participants; those with abnormal results were informed accordingly and referred to the nearest health care centers for appropriate management.

## Results

Of the 998 adult (18 to 55years) participants screened, 229 were excluded; 25 (2.5%) due to missing data, (14 had no demographic information, 11 had no laboratory information), 139 (13.9%) were HIV positive, 3 (0.3%) had unknown HIV status, 38 (3.8%) were HBsAg positive and 24 (2.4%) had no HBsAg results. Complete data from 769 adults were included in this analysis. Of these, 588 (76.5%) were from Harare, 128 (16.6%) from Chitungwiza and 53 (6.9% from Mutoko). The median age of the participants was 28 [IQR: 23–35] years and 412 (54%) were male. There were 242 (98 females, 144 males) in the 18–24 years age-band; 328 (139 females, 189 males) in the 25–34 years age-band; 148 (86 females, 62 males) in the 35–44 years age-band and 51 (34 females, 17 males) in the 44–55 years age- band. The majority of the participants had attained at least secondary education level, 706 (91.8%).

### Hematological indices

The median values and 2.5^th^—97.5^th^ percentile ranges of hematology parameters stratified by gender are presented in Table 1.

**Table 1:** Hematology, 95% Reference Intervals.

Analyte/Unit	RI: Female				RI: Male				RI: Combined				Current in Use
	n	Median	Mean(sd)	Range	n	Median	Mean(sd)	Range	n	Median	Mean(sd)	Range	
*WBC x10^3^ cells/μL	357	5.2	5.4(1.3)	3.3–8.3	412	4.6	4.85(1.25)	2.8–8.1	769	4.9	5.1(1.2)	2.9–7.9	4.0–11.0
*RBC x10^6^ cells/μL	357	4.7	4.8(0.5)	3.9–5.9	412	5.5	5.5(0.6)	4.4–6.7	769	5.1	5.2(0.6)	4.1–6.6	3.8–5.8
*HB g/dL	357	13.5	13.4(1.4)	10.2–15.9	412	15.9	15.8(1.3)	13.2–18.3	769	14.8	14.7(1.8)	10.5–18.0	11.5–16.5
*HCT %	357	41.7	41.7(3.6)	33.9–48.7	412	48.5	48.5(3.4)	42.0–55.1	769	45.4	45.3(4.9)	34.7–54.2	37.0–47.0
MCV fL	357	88.2	87.4(7.4)	68.8–100.7	412	88.8	88.7(7.1)	72.8–102.6	769	88.5	88.1(7.4)	70.8–102.2	77.0–93.0
*MCH, pg	357	28.4	28.0(2.8)	20.7–32.1	412	29.4	29.0(2.5)	22.9–33.5	769	28.9	28.5(2.7)	21.9–32.9	27.0–32.0
*MCHC, g/dL	357	32.1	32.0(1.3)	29.2–34.3	412	32.7	32.7(1.4)	29.8–35.4	769	32.4	32.4(1.4)	29.5–35.0	31.0–35.0
Red Cell Width, %	357	43.1	43.8(3.9)	37.3–52.7	412	43.1	43.8(3.84)	37.7–53.1	769	43.2	44.1(4.4)	37.5–55.4	-
*PLT x10^3^ cells/μL	357	268.5	276.3(64.4)	163.8–431.0	412	229.0	231.2(55.9)	125.3–357.0	769	247	251.1(62.9)	138–384.8	150–400
MPV, fL	357	10.2	10.3(0.9)	8.7–12.3	411	10.2	10.3(0.9)	8.7–12.4	769	10.2	10.3(0.9)	8.7–12.3	-
Neutrophil count %	357	45.3	44.9(9.0)	27.1–62.0	412	43.3	43.6(10.3)	22.1–62.8	769	44.6	44.2(9.9)	25.7–62.4	-
*Neutrophil count cell/mm^3^	357	2272.1	2449.1(905.5)	1112.0–4440.1	412	1997.6	2111.9(820.7)	772.7–3967.3	769	2117.9	2281.3(894.1)	939.2–4252.3	2000–7500
Lymphocyte count %	357	43.1	43.1(7.9)	28.4–59.0	412	43.0	42.8(9.5)	24.1–60.3	769	43	43(8.4)	26.3–59.3	-
*Lymphocyte count cell/mm^3^	357	2149	2292.1(607.5)	1339.6–3737.5	412	1998.5	2044.1(542.3)	1144.3–3276.0	769	2079	2155.9(577.5)	1200.5–3491.6	1500–4000
*Monocyte count %	357	7.6	7.8(1.9)	4.6–12.1	412	8.6	8.9(2.4)	5.1–15.0	769	8.1	8.5(2.4)	4.8–14.4	-
Monocyte count cell/mm^3^	357	392	419.3(132.3)	211.0–723.6	412	409	428.9(142.5)	212.0–795.3	769	400	424.2(137.8)	212.2–766.9	200–800
Eosinophil count %	357	2.3	3.3(2.7)	0.4–10.8	412	2.7	3.9(3.6)	0.4–15.4	769	2.4	3.6(3.2)	0.4–12.9	-
Eosinophil Count cell/mm^3^	357	130	175.1(154.3)	18.7–622.8	412	119	176.2(159.6)	19.0–612.0	769	120	173.5(153.5)	19–611	40–400
*Basophil count %	357	0.4	0.4(0.2)	0.1–1.0	412	0.4	0.5(0.3)	0.1–1.3	769	0.4	0.4(0.3)	0.1–1.2	-
**Basophil count cell/mm^3^**	**357**	**20**	**21.3(12.1)**	**7.0–51.4**	**412**	**20**	**22.5(13.5)**	**8.0–53.0**	**769**	**20**	**22(12.9)**	**8–53**	**10–100**

*parameters with statistically significant gender differencesWBC-White Blood Cell Count; RBC-Red Blood Cell Count; HB-Hemoglobin; HCT-Hematocrit; MCV-Mean Corpuscular Volume; MCH-Mean Corpuscular Hemoglobin; MCHC-Mean Corpuscular Hemoglobin Concentration; PLT-Platelet Count; MPV-Mean Platelet Volume.

Compared with women, men had significantly higher red cell count, hemoglobin, hematocrit, and mean corpuscular hemoglobin while women had significantly higher white cell count, platelets, absolute neutrophil count and absolute lymphocyte counts. The median WBC was 4.6 ×10^3^ cells/μL and 5.2 × 10^3^ cells/ μL for males and females respectively (p<0.001). The combined WBC reference range was 2.9–7.9 × 10^3^ cells /μL. The reference range for hemoglobin was 13.2–18.3 g/dL for males and 10.2–15.9 g/dL for females with significantly different medians of 15.9 g/dL and 13.5 g/dL, respectively (p<0.001). The median platelet count for males was 229.0× 10^3^ cells/μL compared to 268.5× 10^3^ cell/μL for females (p<0.001). The combined reference range was 138–384.8 ×103 cell/μL. The lower limit of neutrophils for males was 772.7 cells/μ/L, reference range 772.7–3967.3 compared to 1112.0 cells/μ/L, reference range 1112.0–4440.1 cells/μL for females with a combined reference range of 939.2 to 4252.3 cells/μ/L.

### Biochemistry

The median values and 2.5^th^—97.5^th^ percentile ranges of hematological parameters stratified by gender are presented in Table 2.

**Table 2:** Chemistry, 95% Reference Intervals.

Analyte/Unit	RI: Female				RI: Male				RI: Combined				Current in Use
	n	Median	Mean(sd)	Range	n	Median	Mean(sd)	Range	n	Median	Mean(sd)	Range	
*Sodium, mmol/L	357	141	141.3(3.1)	135–148	412	143	142.7(3.2)	136–149	769	142	142(3.2)	136–149	133–146
*Potassium, mmol/L	357	4.2	4.3(0.4)	3.5–5.2	412	4.4	4.4(0.4)	3.6–5.3	769	4.3	4.3(0.4)	3.6–5.2	3.5–5.2
*Chloride, mmol/L	357	102	102(2.9)	96–107	412	101	101(4.5)	95–107	769	101	101(2.9)	96–107	96–109
*Bicarbonate, mmol/L	357	22.4	22.5(3.1)	16.1–29.1	412	24.3	24.5(3.0)	18.8–31.6	769	23.4	23.6(3.2)	17.2–30.9	21–29
*Urea,mg/dL	357	8.7	8.9(2.7)	3.9–15.4	412	9.8	9.9(2.8)	4.8–15.4	769	9.3	9.5(2.9)	4.1–15.4	5.6–18.8
*Creatinine, mg/dL	357	0.7	0.7(0.1)	0.5–1.1	412	0.9	0.9(0.2)	0.7–1.3	769	0.8	0.8(0.2)	0.6–1.2	0.5–1.5
*ALP, IU/L	357	69	72.6(23.1)	39–131	412	80	84.2(24.4)	49–149	769	75	78.7(24.5)	42–144.3	34–140
*ALT, IU/L	357	15	16.9(7.5)	5–35	412	21	23.5(11.9)	9.0–58.8	769	18	20.5(10.4)	7–51	5–44
*AST, IU/L	357	23	23.3(6.3)	12.0–40.0	412	28.0	30.3(9.8)	17.0–57.0	769	25	25.8(6.7)	14–41	10–30
*GGT, IU/L	357	20	22.9(10.3)	8.0–51.0	412	25	27(11.4)	10.5–58.1	769	23	25.3(11.5)	10–57	13–60
*T. Bilirubin, mg/dL	357	0.5	0.5(0.2)	0.2–1.1	412	0.6	0.7(0.3)	0.3–1.6	769	0.6	0.64(0.3)	0.3–1.5	0.2–1. 7
*D. Bilirubin, mg/dL	357	0.1	0.1(0.01)	0.0–0.3	412	0.1	0.2(0.1)	0.0–0.4	769	0.1	0.1(0.1)	0.0–0.4	0.0–0.2
*Total Protein, g/L	357	7.8	7.9(0.6)	6.8–9.4	412	8	8.1(0.6)	71–93	769	7.9	8.0(0.6)	6.9–9.3	5.0–8.6
* Albumin, g/L	357	4.7	4.8(3.9)	4.1–5.5	412	5	5.1(0.3)	4.5–5.9	769	4.9	4.9(0.4)	4.2–5.7	3.5–5.2
*Gluc. random, mg/dL	357	78	78.4(9.2)	63.5–99.0	412	79.5	79.7(9.4)	61–103	769	79	79.1(9.5)	62–101.7	59–141
*Calcium, mmol/L	357	9.1	9.1(0.7)	7.9–10.8	412	9.4	9.5(0.7)	8.2–11.4	769	9.3	9.3(0.7)	8.1–11.2	8.2–10.2
Phosphate mg/dL	357	3.4	3.4(0.6)	2.3–4.7	412	3.3	3.3(0.6)	2.3–4.5	769	3.3	3.4(0.6)	2.3–4.6	2.4–4.4
*Creat. Kinase, IU/L	357	149	168.7(73.7)	76–389.8	412	197.5	221.9(101.5)	83.5–504.5	769	173	196.5(89.9)	76–437.2	F: 0.0–145M: 0.0–171
*Lipase, IU/L	357	32	36.7(15.9)	16.7–81.0	412	30	32.6(12.2)	15.0–62.7	769	31	34.3(13.9)	16–67	7–58
*Amylase, IU/L	357	90	93.5(29.4)	43.9–159.6	412	102	107(33.6)	53.2–186.7	769	96	100.8(31.8)	49–176	0.0–100
T. Chol./HDL Ratio	357	3.3	3.4(0.9)	2.1–5.6	412	3.0	3.1(0.9)	1.7–5.4	769	3.1	3.2(0.9)	1.8–5.6	-
Triglycerides, mg/dL	357	68	75.5(33)	33.8–159.5	412	69	76.1(30.8)	37.0–156.1	769	69	75.3(31)	35–155.4	40–136
*Total Chol.,mg/dL	357	163	166.2(35.2)	100.9–240.0	412	149	152.4(33.7)	99–232	769	156	159.1(35.6)	99.1–237	0–200
LDL-Chol., mg/dL	357	100	102(28.6)	52–160	412	82	85.5(29.3)	40.9–159.3	769	90	92.5(28.5)	47–154.3	<131
**HDL-Chol., mg/dL**	**357**	**49.5**	**51.9(13.3)**	**31.9–85.0**	**412**	**49**	**51.4(14.1)**	**31–87**	**769**	**49**	**51.7(13.7)**	**31–85**	**>39**

*parameters with statistically significant gender differences

ALP-Alkaline Phosphatase; ALT-Alanine Aminotransferase; AST-Aspartate Aminotransferase, GGT-Gama Glutamyl transferase

T. Bilirubin-Total Bilirubin; D. Bilirubin-Direct Bilirubin; Gluc. Random- Random Glucose; Creatinine Kinase; Total Chol-Total Cholesterol; LDL-Chol-Low Density Lipoprotein Cholesterol; HDL-Chol-High Density Lipoprotein Cholesterol.

We established chemistry reference ranges for 24 analytes; renal function tests (urea, creatinine), serum electrolytes (ALP, ALT, AST, total bilirubin, direct bilirubin, total protein, albumin, GGT), non-fasting lipid profile [triglycerides, total cholesterol, LDL cholesterol, HDL cholesterol), pancreatic enzymes, (lipase, amylase), creatinine kinase and random glucose. Males had higher levels of urea, sodium, potassium, calcium, creatinine, amylase, total protein, albumin, and liver enzymes levels compared to females (p<0.001). Females had higher total cholesterol and LDL-cholesterol compared to males. The median total cholesterol for males was 149 mg/dL compared to 163 mg/dL for females (p<0.001). The combined reference range was 99.1–237 mg/dL. The median LDL-cholesterol values were 82 mg/dL for males and 100mg/dL for females (p<0.001). No sex differences were observed for HDL-cholesterol and triglycerides. Creatinine kinase was significantly higher in males with a median of 197.5 IU/L compared to 149 IU/L in females. The combined reference range was 76–437.2 IU/L.

## Discussion

This is the first population-based study in Zimbabwe to comprehensively study a wide range of hematological and biochemical laboratory indices using internationally accepted CLSI guidelines [11]. Results from this study showed several differences with the existing reference ranges. Reference ranges for WBC, neutrophil counts and platelets reported in this study were noted to be lower than those currently in use at the UZ-UCSF (for patient care and research). LDL cholesterol, HDL cholesterol and direct bilirubin from this study were higher compared to the existing reference ranges. We observed very high creatinine kinase reference range of 76–437 IU/L compared to the current range of 0–145 IU/L. Our study also showed higher lipase and amylase compared to the existing reference ranges.

The reference range values for hemoglobin, hematocrit, and red blood cells are comparable to those established by the International AIDS Vaccine Initiative (IAVI) at seven clinical centers in four sub-Saharan African countries; Rwanda, Uganda, Kenya and Zambia [14]. Several hematological indices including hemoglobin, hematocrit, and red blood cell count reference ranges for both males and females from our study are comparable to those from previous African studies [8, 15, 16, 17]. The reference range for neutrophils for males in our study was 773–3967 which was lower than that observed among South Africans, Tanzanian and Ugandan males [15, 16, 18]. However our female reference ranges were comparable to those of other African countries [16, 17].

The finding of low white cell counts and low neutrophil counts among African populations has been frequently reported and thought to be constitutional [1,2,19,20]. Our data showed higher platelet counts in females, a finding previously reported in other studies [1, 21]

Higher reference ranges for urea, sodium, potassium, calcium and creatinine in males compared to females have been reported in studies done in Tanzania, Uganda and Kenya [16, 18, 22]. High creatinine in males compared to females is expected and in many international settings is explained by a greater skeletal, muscle and bone mass in males [23].

Combined male and female reference ranges for sodium, potassium and chloride were comparable to those reported in an Ethiopian study [24]. However we found higher phosphate ranges than those from this study.

Total cholesterol, HDL cholesterol and LDL cholesterol levels were higher when compared with results from Tanzanian and Kenyan, studies [16, 22]. In comparing males and females our study showed higher HDL cholesterol in females than males similar to findings in a Botswana study [25].

In using reference ranges to grade adverse events in international research settings we have observed occasional differences between ranges provided by the sponsor and those of the local population. In our setting the UZ-UCSF implements NIH sponsored studies and uses DAIDS (Division of AIDS, National Institute of Allergy and Infectious Diseases, NIH) toxicity grading scales to identify possible adverse events [26]. The DAIDS toxicity table grades ANC as grade 1 (1000–1300 cells/mm^3^), grade 2 (750–999 cell/mm^3^), grade 3 (500-749/mm^3^) and grade 4 (<500 cells/mm^3^). A male in our setting with a neutrophil count of 773 cells/mm^3^ would be regarded as having a grade 2 adverse event yet this is normal according to the reference ranges we have established in this study. Moreover the lower limit of the combined neutrophil reference range of 939 cells/ mm^3^ would still be a grade 2. Similar discrepancies are also observed in cholesterol and phosphate. In the research setting this discrepancy between normal references ranges in our population and the toxicity levels provided in the sponsors table may impact patient management.

One of the limitations of our study is that the glucose and lipid samples were non- fasting. Most references values for serum lipids are established on fasting blood samples when used for cardiovascular risk assessment according to most national and international guidelines [27]. Some studies suggest that the variation between fasting and non-fasting LDL-Cholesterol levels is small [27, 28]. Another limitation is that we did not screen for other infections like malaria and helminths which could impact hematological indices such as hemoglobin levels and eosinophil counts respectively.

## Conclusions

We conclude that these reference values are contemporary and represent an improvement on how the previous values were obtained. We suggest that these reference ranges can be immediately adopted for use in the clinical care of patients in our institutions including use in research.
